# Fasting blood glucose level and risk of all‐cause and cause‐specific mortality in peritoneal dialysis patients

**DOI:** 10.1111/1753-0407.13601

**Published:** 2024-09-12

**Authors:** So Jin Lim, Ju Young Moon, Kyung Hwan Jeong, Gang Jee Ko, Yun Jin Choi, Hyeon Seok Hwang

**Affiliations:** ^1^ Division of Nephrology, Department of Internal Medicine Kyung Hee University Medical Center, Kyung Hee University, College of Medicine Seoul Republic of Korea; ^2^ Division of Nephrology, Department of Internal Medicine Korea University, College of Medicine Seoul Republic of Korea; ^3^ Biomedical Research Institute Korea University Guro Hospital Seoul Republic of Korea

**Keywords:** all‐cause mortality, cause‐specific mortality, diabetes, fasting blood glucose, peritoneal dialysis

## Abstract

**Background:**

Glycemic control is crucial in peritoneal dialysis (PD) patients with diabetes. Although fasting blood glucose (FBG) is the most commonly used index to measure blood glucose levels, there is currently no evidence supporting the association between FBG level and mortality risk in PD patients.

**Methods:**

A total of 3548 diabetic PD patients between 2002 and 2018 were enrolled from the National Health Insurance Service database of Korea. We investigated the association between FBG levels and the risk of all‐cause and cause‐specific mortality.

**Results:**

Patients with FBG levels 80–99 mg/dL exhibited the highest survival rates, whereas those with FBG levels ≥180 mg/dL had the lowest survival rates. Compared with FBG levels 80–99 mg/dL, the adjusted hazard ratios and 95% confidence interval for all‐cause mortality significantly increased as follows: 1.02 (0.87–1.21), 1.41 (1.17–1.70), 1.44 (1.18–2.75), and 2.05 (1.73–2.42) for patients with FBG 100–124 mg/dL, FBG 125–149 mg/dL, FBG 150–179 mg/dL, and FBG ≥180 mg/dL, respectively. The risk for all‐cause mortality also showed an increasing pattern in patients with FBG levels <80 mg/L. The risk of cardiovascular death significantly increased as FBG levels exceeded 125 mg/dL. However, the risk of infection‐related and malignancy‐related deaths did not show a significant increase with increasing FBG levels.

**Conclusion:**

There was an increase in the risk of all‐cause mortality as FBG levels exceeded 125 mg/dL in PD patients with diabetes, and the risk of cardiovascular death showed a strong correlation with FBG levels compared with other causes of death.

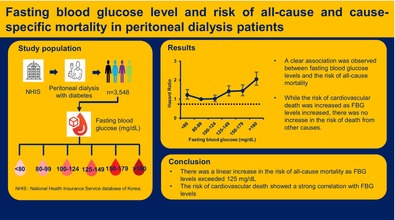

## Graphical abstract

Highlights

- A clear association was observed between FBG levels and the risk of all‐cause mortality in PD patients, indicating that FBG is a significant indicator for quantifying the risk of all‐cause mortality in these patients.
- Although a higher FBG level was significantly associated with an increased risk of cardiovascular‐related death, there was no notable increase in the risk of death from cancer or infections as the FBG levels increased.

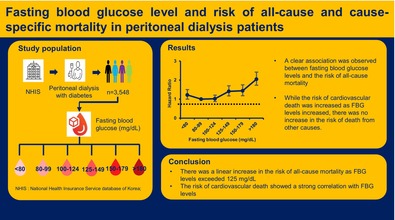

## 1 INTRODUCTION

Diabetes is the leading cause of end‐stage renal disease worldwide,^1^ and the number of patients with diabetes undergoing dialysis is constantly increasing.^2^, ^3^ Diabetes has serious clinical implications for patients undergoing dialysis, and uncontrolled hyperglycemia induces metabolic derangements and organ injuries.^4^, ^5^ Several studies have indicated that patients with diabetes undergoing dialysis have a worse prognosis compared with those having no diabetes, and proper glycemic control is critical for the improvement of clinical prognosis.^3^, ^6^, ^7^ Indeed, a number of studies have reported that achieving target glucose control is a pivotal factor in decreasing the risk of cardiovascular complications and all‐cause mortality.^8^, ^9^, ^10^, ^11^

Glycemic control is highly important in peritoneal dialysis (PD) patients. Dialysis solutions used in PD patients contain high concentrations of glucose, and obligatory glucose absorption from these solutions contributes to hyperglycemia.^12^, ^13^ Fasting blood glucose (FBG) reflects basal glucose load and is an indicator of the daily status of hyperglycemia.^14^, ^15^ It helps determine or plan antidiabetic management as the starting point for daily glycemic change. Additionally, several studies have indicated that FBG level is the key factor in determining both cardiovascular complications and the risk of all‐cause mortality.^16^, ^17^, ^18^, ^19^, ^20^ Although the advantages of monitoring FBG levels make them a crucial component for achieving optimal glycemic control in patients who do not require dialysis, the information on the optimal FBG level required to reduce the risk of mortality is lacking. Furthermore, there are few studies on the association between FBG levels and mortality risk in PD patients with diabetes.

Therefore, we investigated the relationship between FBG levels and the risk of all‐cause mortality in diabetic PD patients. Additionally, we aimed to evaluate the association between FBG levels and the risk of cause‐specific deaths to identify which causes of death are most influenced by FBG levels.

## 2 MATERIALS AND METHODS

### 2.1 Study population

In this study, we used the National Health Insurance Database (NHID), a publicly available database that contains information on healthcare utilization, sociodemographic factors, and mortality for the entire population of South Korea. The National Health Insurance Service (NHIS) accumulates complete data on all medical service claims and maintains databases.^21^ The NHIS also operates the National Health Screening Examination Program, which includes medical interviews, anthropometric examinations, laboratory tests, and others. We set the inclusion criteria as follows: (1) subjects over the age of 19 years; (2) subjects who received PD and National Health Screening examinations from 2002 to 2018; (3) subjects with type 2 diabetes. PD patients were defined as those receiving PD with the PD‐specific insurance code (V003) more than twice and for more than 1 month, and they were matched to those who underwent the National Health Screening examination. The presence of diabetes was defined according to the following criteria: at least one claim per year under the *International Classification of Diseases*, *Tenth Revision* (*ICD‐10*) codes (E11–14) and at least 30 days of claim per year for the prescription of antidiabetic medication. A total of 3663 patients met the inclusion criteria, and individuals were excluded from this study if they had incomplete information (*n* = 115). Therefore, 3548 patients undergoing PD with diabetes were included in this study (Figure S1). All patients were followed up from the initial health screening to the date of death, date of their last checkup, or until December 31, 2018. The institutional review board approved this study (no. 1607–187‐779), and the usage of the NHIS database was also approved (no. 2018–1‐148). The study protocol complied with the principles of the Declaration of Helsinki.

### 2.2 Data collection

The following baseline demographic and clinical characteristics were collected: age, sex, height, weight, body mass index (BMI), systolic blood pressure (SBP), dialysis duration, and Charlson Comorbidity Index. The laboratory measurements included FBG, hemoglobin level, and lipid profiles. Blood samples were collected after 8 h of fasting. Blood pressure was measured at least twice using mercury or an automatic sphygmomanometer after a minimum rest period of 5 min with the individual in a sitting position. The Charlson Comorbidity Index score is a measure of the burden of comorbidities; it was calculated using *ICD‐10* codes.^22^ FBG was analyzed as a categorical variable, with FBG levels divided into six categories based on previous reports: <80 mg/dL, 80–99 mg/dL, 100–124 mg/dL, 125–149 mg/dL, 150–179 mg/dL, and ≥180 mg/dL.^23^, ^24^, ^25^, ^26^ Patients were categorized for subgroup analysis based on predefined criteria, including age, sex, Charlson Comorbidity Index, and hemoglobin levels. These criteria are primary risk factors for all‐cause mortality in PD patients and are considered factors when determining the FBG target.^23^, ^27^, ^28^

### 2.3 Outcomes

The primary outcome was all‐cause mortality during follow‐up. The secondary outcome was association between FBG and the risk of cause‐specific mortality. Mortality data were collected from the NHIS database using death certificates. Computerized searches of the death certificate data from the National Statistical Office in Korea were performed for each mortality case. Cause‐specific mortality was investigated by dividing the data into three categories: cardiovascular death, malignancy‐related death, and infection‐related death. Deaths with *ICD‐10* codes I00–I99 were counted as cardiovascular cause, and A00–B99 and J10–18 were considered as infectious cause. Malignancy‐related death was categorized with C00–99 codes.

### 2.4 Statistical analyses

Continuous variables were presented as mean ± standard deviation and compared using a one‐way analysis of variance. Categorical variables were analyzed using the chi‐square test. Kaplan–Meier analysis was used to compare patient survival rates across different FBG levels. The association between FBG levels and mortality risk was assessed using a Cox‐proportional hazards model with adjustments for age, sex, BMI, dialysis duration, Charlson Comorbidity Index, SBP, hemoglobin level, and total cholesterol level. The presence of crossovers in Kaplan–Meier curves between different FBG categories was examined to assess the proportional hazards assumption. Statistical significance was set at *p* < 0.05. All analyses were performed using the SAS Enterprise guide 7.1 software.

## 3 RESULTS

### 3.1 Baseline characteristics

Table 1 shows the baseline characteristics of included patients; the mean age was 53.9 ± 11.3 years, and 62.8% of them were men. The number of participants in each FBG level was 353 (9.9%), 952 (26.8%), 896 (25.3%), 440 (12.4%), 362 (10.2%) and 545 (15.4%) for FBG <80 mg/dL, 80–99 mg/dL, 100–124 mg/dL, 125–149 mg/dL, 150–179 mg/dL, and ≥180 mg/dL, respectively. The age of PD patients increased as their FBG levels increased, and higher FBG levels were also associated with increased BMI and Charlson Comorbidity Index scores. Patients with FBG levels ≥180 mg/dL had lower hemoglobin levels and higher total cholesterol levels than those with lower FBG levels.

**TABLE 1:** Baseline characteristics of subjects.

Variable	FBG categories (mg/dL)						
	<80 (*n* = 353)	80–99 (*n* = 952)	100–124 (*n* = 896)	125–149 (*n* = 440)	150–179 (*n* = 362)	≥180 (*n* = 545)	*p* value
Age (year)	52.7 ± 11.9	52.0 ± 12.5	54.7 ± 11.0	55.4 ± 10.2	54.8 ± 10.3	54.5 ± 10.0	<0.001
Male sex	224 (63.5)	583 (61.2)	598 (66.7)	293 (66.6)	227 (62.7)	304 (55.8)	<0.001
BMI (kg/m^2^)	23.8 ± 3.5	23.8 ± 3.5	24.1 ± 3.4	24.2 ± 3.3	24.3 ± 3.5	24.4 ± 3.8	0.011
SBP (mmHg)	134.7 ± 20.1	134.5 ± 20.1	134.5 ± 19.9	136.8 ± 20.7	135.0 ± 20.5	135.7 ± 19.9	0.387
Dialysis duration (year)	3.1 ± 3.0	3.3 ± 3.0	3.2 ± 3.0	2.9 ± 2.9	3.1 ± 2.9	2.9 ± 2.6	0.140
CCI	5.6 ± 1.8	5.1 ± 1.9	5.4 ± 1.9	5.7 ± 1.7	6.0 ± 1.7	6.1 ± 1.7	<0.001
FBG (mg/dL)	72.0 ± 7.6	90.6 ± 5.5	110.4 ± 7.0	135.7 ± 7.1	162.8 ± 8.6	246.8 ± 69.7	<0.001
Hemoglobin (g/dL)	11.4 ± 1.9	11.3 ± 2.1	11.3 ± 1.9	11.3 ± 1.9	11.1 ± 1.7	10.9 ± 1.8	<0.001
TC (mg/dL)	176.4 ± 49.3	184.7 ± 81.0	183.2 ± 49.2	179.6 ± 47.8	182.3 ± 53.3	189.7 ± 60.5	0.028
HDL‐C (mg/dL)	47.7 ± 31.2	49.1 ± 19.1	48.3 ± 29.3	44.6 ± 14.8	45.6 ± 20.0	46.6 ± 28.7	0.032
LDL‐C (mg/dL)	95.5 ± 37.1	100.6 ± 35.8	99.4 ± 37.7	97.9 ± 38.9	99.7 ± 39.7	99.1 ± 45.1	0.485

Abbreviations: BMI, body mass index; CCI, Charlson Comorbidity Index; FBG, fasting blood glucose; HDL‐C, high‐density lipoprotein‐cholesterol; LDL‐C, low‐density lipoprotein‐cholesterol; SBP, systolic blood pressure; TC, total cholesterol.

### 3.2 FBG and all‐cause mortality

**FIGURE 1:**
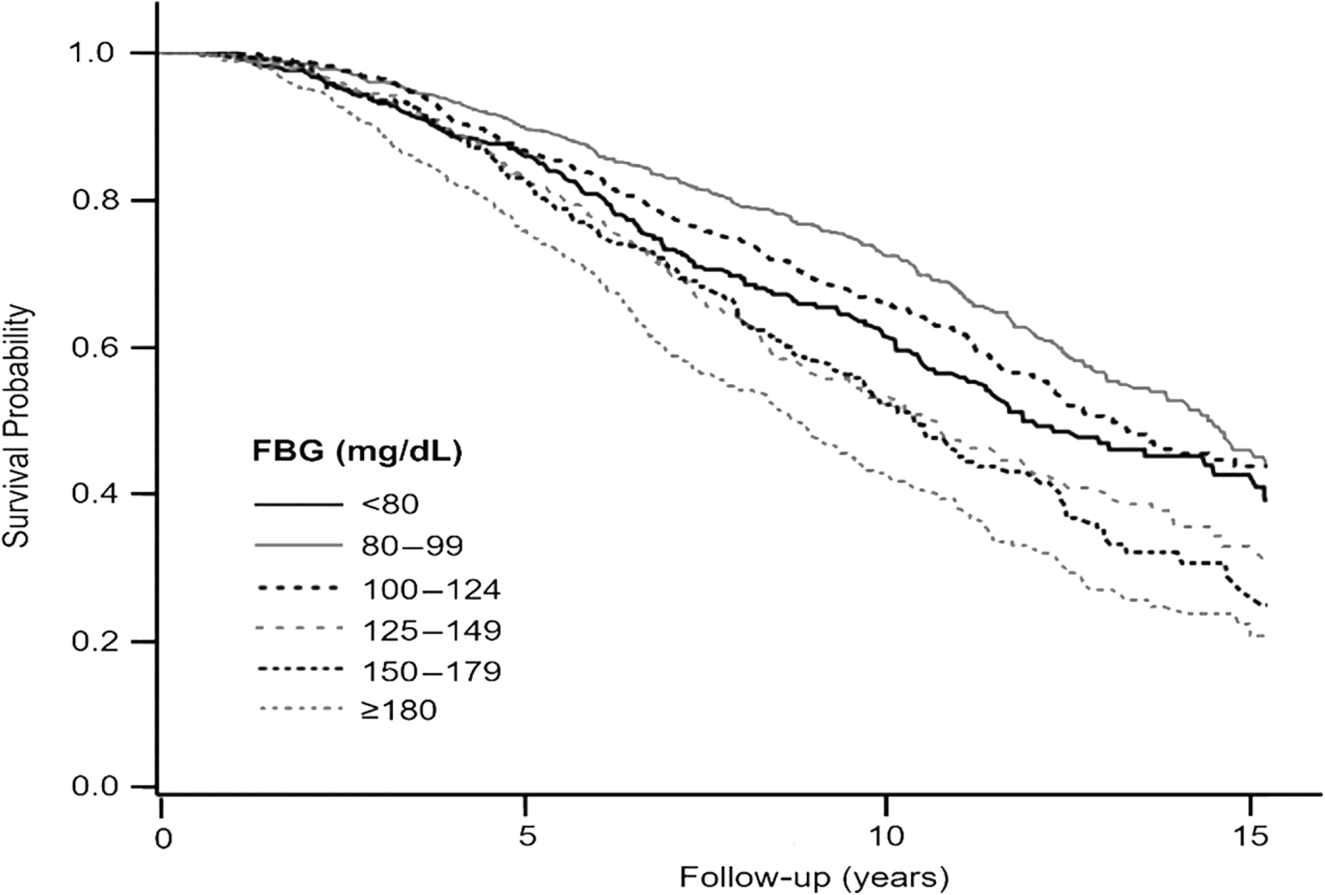
Kaplan–Meier curves for mortality according to FBG levels. FBG, fasting blood glucose.

During a median follow‐up period of 6.55 years, 1366 mortality events were identified, and patient survival rates differed significantly across FBG levels (Figure 1). Patients with FBG levels ranging from 80 to 99 mg/dL exhibited higher survival rates than those with other FBG levels, and patients with FBG levels ≥180 mg/dL had the lowest survival rates (*p* < 0.001). Table 2 presents the association between FBG levels and the risk of all‐cause mortality. In the univariate analysis, all FBG levels ≥100 mg/dL showed an increased risk of mortality compared with FBG levels of 80–99 mg/dL. FBG levels <80 mg/dL were also associated with a higher risk of mortality. In the multivariate Cox regression analysis, the association between higher FBG levels and increased mortality risk remained significant. In comparison with FBG levels of 80–99 mg/dL, the hazard ratios (HRs) of all‐cause mortality were 1.02 (95% confidence interval [CI], 0.87–1.21; *p* = 0.781), 1.41 (95% CI, 1.17–1.70; *p* < 0.001), and 1.44 (95% CI, 1.18–1.75; *p* < 0.001) for FBG levels of 100–124, 125–149, and 150–179 mg/dL, respectively. The FBG levels ≥180 mg/dL were associated with the highest risk of mortality (HR, 2.05; 95% CI, 1.73–2.42; *p* < 0.001). Patients with FBG levels <80 mg/dL showed an increasing pattern of HR than those with FBG levels of 80–99 mg/dL (HR, 1.22; 95% CI, 1.00–1.50; *p* = 0.056).

**TABLE 2:** Incidence and HR of FBG levels for all‐cause mortality.

FBG (mg/dL)	No. of event (%)	Person‐years	Incidence rate	Univariate		Multivariate	
				HR (95% CI)	*p* value	HR^a^ (95% CI)	*p* value
<80	142 (40.2)	2865	49.6	1.39 (1.13–1.70)	0.002	1.22 (1.00–1.50)	0.056
80–99	277 (29.1)	7827	35.4	Reference	–	Reference	–
100–124	306 (34.2)	7192	42.5	1.22 (1.04–1.44)	0.015	1.02 (0.87–1.21)	0.781
125–149	192 (43.6)	3301	58.2	1.73 (1.44–2.08)	<0.001	1.41 (1.17–1.70)	<0.001
150–179	164 (45.3)	2666	61.5	1.84 (1.51–2.23)	<0.001	1.44 (1.18–1.75)	<0.001
≥180	285 (52.3)	3712	76.8	2.41 (2.05–2.85)	<0.001	2.05 (1.73–2.42)	<0.001

Abbreviations: BMI, body mass index; CI, confidence interval; FBG, fasting blood glucose; HR, hazard ratio; No., number.

^a^ Adjusted for age, sex, BMI, dialysis duration, Charlson Comorbidity Index, systolic blood pressure, total cholesterol, and hemoglobin.

### 3.3 Subgroup analysis

In subgroup analyses, we calculated HRs of FBG levels in different subgroups based on the predefined criteria of age, sex, Charlson Comorbidity Index, and hemoglobin (Table 3). The analysis revealed that all subgroups exhibited a significant increase in the risk of all‐cause mortality when FBG levels reached ≥100 mg/dL. Furthermore, among patients with a hemoglobin level ≥10 g/dL, the HR for all‐cause mortality was significantly increased for those with FBG <80 mg/dL. However, in other subgroups, a notable increase in the risk of all‐cause mortality was not observed in patients with FBG <80 mg/dL.

**TABLE 3:** Risk of FBG levels for the all‐cause mortality based on predefined subgroups.

	Subgroup	FBG (mg/dL)	No. of events (%)	HR (95% CI)	*p* value	HR^a^ (95% CI)	*p* value
Age	<65 years	<80	103 (72.5)	1.55 (1.22–1.98)	<0.001	1.26 (0.99–1.62)	0.063
		80–99	177 (63.9)	Reference	–	Reference	–
		≥100	676 (71.4)	1.88 (1.59–2.22)	<0.001	1.56 (1.32–1.85)	<0.001
	≥65 years	<80	39 (27.5)	1.13 (0.78–1.65)	0.518	1.11 (0.75–1.63)	0.602
		80–99	100 (36.1)	Reference	–	Reference	–
		≥100	271 (28.6)	1.36 (1.08–1.72)	0.009	1.34 (1.06–1.69)	0.014
Sex	Male	<80	101 (71.1)	1.48 (1.16–1.88)	0.001	1.22 (0.96–1.56)	0.106
		80–99	193 (69.7)	Reference	–	Reference	–
		≥100	627 (66.2)	1.56 (1.33–1.84)	<0.001	1.27 (1.08–1.50)	0.004
	Female	<80	41 (28.9)	1.19 (0.81–1.73)	0.373	1.13 (0.77–1.67)	0.536
		80–99	84 (30.3)	Reference	–	Reference	–
		≥100	320 (33.8)	1.90 (1.49–2.41)	<0.001	1.57 (1.23–2.00)	<0.001
CCI	<6	<80	64 (45.1)	1.33 (0.99–1.78)	0.056	1.33 (0.99–1.79)	0.056
		80–99	157 (56.7)	Reference	–	Reference	–
		≥100	376 (39.7)	1.48 (1.23–1.79)	<0.001	1.24 (1.02–1.49)	0.027
	≥6	<80	78 (54.9)	1.31 (0.98–1.74)	0.067	1.23 (0.92–1.64)	0.162
		80–99	120 (43.3)	Reference	–	Reference	–
		≥100	571 (60.3)	1.63 (1.34–1.99)	<0.001	1.57 (1.29–1.91)	<0.001
Hb	<10 g/dL	<80	35 (24.7)	1.43 (0.96–2.13)	0.083	1.13 (0.75–1.71)	0.732
		80–99	75 (27.1)	Reference	–	Reference	–
		≥100	259 (27.4)	1.80 (1.39–2.33)	<0.001	1.55 (1.19–2.01)	0.001
	≥10 g/dL	<80	107 (75.4)	1.39 (1.10–1.76)	0.006	1.28 (1.01–1.62)	0.044
		80–99	202 (72.9)	Reference	–	Reference	–
		≥100	688 (72.7)	1.63 (1.39–1.91)	<0.001	1.30 (1.11–1.52)	0.001

Abbreviations: BMI, body mass index; CCI, Charlson Comorbidity Index; CI, confidence interval; FBG, fasting blood glucose; Hb, hemoglobin; HR, hazard ratio; No, number.

^a^ Adjusted for age, sex, BMI, dialysis duration, CCI, systolic blood pressure, total cholesterol, and Hb.

### 3.4 Cause‐specific death analysis

Mortality risk of cardiovascular deaths, malignancy‐related deaths, and infection‐related deaths are shown in Table 4. During the follow‐up period, cardiovascular death occurred in 252 (7.1%) patients, malignancy‐related death in 86 (2.4%), and infection‐related death in 71 (2.0%). The risk of cardiovascular death was significantly increased with increasing FBG levels. Compared with those having FBG 80–99 mg/dL, cardiovascular mortality risk was 1.11 (95% CI, 0.76–1.61; *p* = 0.587), 1.56 (95% CI, 1.03–2.37; *p* = 0.037), 1.70 (95% CI, 1.10–2.63; *p* = 0.016), and 1.56 (95% CI, 1.02–2.38; *p* = 0.040) in patients having FBG levels of 100–124, 125–149, 150–179, and ≥180 mg/dL, respectively. The risk of malignancy‐related deaths and infection‐related deaths were not significantly increased with increasing FBG levels.

**TABLE 4:** Incidence and HR of FBG levels for cause‐specific mortality.

	FBG categories (mg/dL)					
Variable	<80	80–99	100–124	125–149	150–179	≥180
CV death						
No. of event (%)	25 (7.1)	52 (5.5)	61 (6.8)	39 (8.9)	35 (9.7)	40 (7.3)
Incidence rate	8.7	6.6	8.5	11.8	13.1	10.8
HR^a^ (95% CI)	1.13 (0.70–1.83)	Reference	1.11 (0.76–1.61)	1.56 (1.03–2.37)	1.70 (1.10–2.63)	1.56 (1.02–2.38)
*p* value	0.620	–	0.587	0.037	0.016	0.040
Malignancy‐related death						
No. of event (%)	9 (2.5)	28 (2.9)	19 (2.1)	11 (2.5)	10 (2.8)	9 (1.7)
Incidence rate	3.1	3.6	2.6	3.3	3.8	2.4
HR^a^ (95% CI)	0.73 (0.34–1.58)	Reference	0.59 (0.33–1.07)	0.82 (0.40–1.66)	1.00 (0.48–2.09)	0.66 (0.30–1.47)
*p* value	0.427	–	0.084	0.574	0.993	0.313
Infection‐related death						
No. of event (%)	9 (2.5)	15 (1.6)	17 (1.9)	10 (2.3)	7 (1.9)	13 (2.4)
Incidence rate	3.1	1.9	2.4	3.0	2.6	3.5
HR^a^ (95% CI)	1.31 (0.57–3.03)	Reference	0.99 (0.49–2.00)	1.36 (0.61–3.04)	1.17 (0.47–2.91)	2.00 (0.94–4.24)
*p* value	0.522	–	0.974	0.458	0.727	0.072

*Note*: Incidence rate is represented as 1000 person‐years.

Abbreviations: BMI, body mass index; CV, cardiovascular; CI, confidence interval; FBG, fasting blood glucose; HR, hazard ratio; No., number.

^a^ Adjusted for age, sex, BMI, dialysis duration, Charlson Comorbidity Index, systolic blood pressure, total cholesterol, and hemoglobin.

## 4 DISCUSSION

This study established a significant relationship between FBG levels and the risk of all‐cause mortality in PD patients with diabetes. The risk of all‐cause mortality increased as FBG levels reached 125 mg/dL or higher, and it seems that the lowest mortality risk was found within the FBG level range of 80–124 mg/dL. Furthermore, a similar association was observed between FBG levels and the risk of cardiovascular death, whereas there was no increase in the risk of death related to malignancy or infections. These findings underscore the critical role of FBG levels as a key factor in predicting both all‐cause and cardiovascular mortality in PD patients.

Intensive blood glucose control is effective in preventing diabetic complications and reducing all‐cause mortality in patients without renal impairment, with a recommended FBG target of 80–130 mg/dL.^29^, ^30^ However, American Diabetes Association recommends that patients with renal impairment need to be less stringent regarding FBG target levels because strict glycemic control increases the risk of hypoglycemia, adverse clinical events, and mortality.^31^ In this study, we expected that the FBG level associated with the lowest mortality would be higher in patients with PD than in those without renal impairment. However, our study suggested that FBG levels between 80 and 124 mg/dL seemed to be associated with the lowest mortality risk in PD patients, and it was similar to the FBG levels in patients without renal impairment. These findings suggest that a blindly upward target of FBG level should be carefully considered to achieve the largest survival benefit.

This study demonstrated a clear association between FBG levels and all‐cause mortality in PD patients, with an increase in mortality risk observed in patients with FBG levels ≥125 mg/dL. These findings suggest that FBG level is a valuable marker for quantifying the adverse effects of hyperglycemia in PD patients. While random glucose levels can be easily measured regardless of food intake status and represent glycemic control levels, a previous study showed that mortality risk significantly increased when random glucose levels were >300 mg/dL.^32^ Therefore, it is suggested that the measurement of FBG levels is more sensitive and efficient than random glucose levels for estimating the risk of hyperglycemia.

Glycated hemoglobin (HbA1c) is the most commonly used method for measuring blood glucose levels in PD patients, and several studies explored the relationship between HbA1c and the risk of all‐cause mortality. Although high HbA1c levels have showed a strong association with an increased risk of all‐cause mortality, the connection between lower HbA1c levels and an elevated risk of mortality has not been as clearly defined.^33^, ^34^, ^35^ In our study, we observed the increasing pattern in the risk of all‐cause mortality in PD patients with FBG levels <80 mg/dL, while the risk of all‐cause mortality did not reach statistical significance. These findings imply that there may be a potential benefit in setting a lower limit for glycemic control based on FBG levels, further supporting the use of HbA1c levels in the management of diabetes in PD patients.

Age and comorbidities substantially affect the association between hyperglycemia and the risk of mortality. Previous research has shown that high HbA1c levels have a greater detrimental effect on the survival rate of younger, male, and nonanemic PD patients, whereas this negative effect is reduced in other PD patients who do not fall into these categories.^32^, ^36^ However, our subgroup analysis revealed that the risk of all‐cause mortality was significantly increased when FBG levels were ≥100 mg/dL, regardless of predefined criteria, suggesting that the association between high FBG levels and mortality risk did not significantly differ among subgroups. These results suggest that patient condition marginally affected the predictability of FBG levels, distinguishing it from the clinical significance of HbA1c in relation to mortality risk.

In cause‐specific mortality study, the association between high FBG and cardiovascular mortality was significant. However, the risk of malignancy‐related or infection‐related death was not significantly increased as FBG levels were increased. These findings suggest that the risk of cardiovascular mortality may respond sensitively to FBG levels than other causes of death in PD patients with diabetes. Therefore, we suggest that proper FBG control can effectively reduce the risk of cardiovascular mortality and emphasize that strict FBG control is considered highly necessary for individuals at high risk for cardiovascular disease. The mechanism elucidating the strong association of FBG and cardiovascular mortality in PD patients was not clear. We presumed that underlying greater burden of cardiovascular disease in PD patients may increase susceptibility to the adverse effects of FBG on the cardiovascular system.

There are several limitations to our study. First, relatively healthy individuals are more likely to receive health checkups, whereas those with multiple comorbidities may not. Therefore, the chance for selection bias exists as we did not enroll patients who did not undergo health checkups. This potential selection bias may have influenced our results. Second, we did not investigate the risk of hypoglycemic events. This information provides further validation for the strict FBG targets, as lower FBG targets are expected to increase the risk of hypoglycemia. Third, different types of medications for diabetes or hypertension may influence the association between FBG level and mortality. However, we did not consider these factors in the mortality analysis. Finally, Kaplan–Meier curves between FBG levels 125–149 mg/dL and 150–179 mg/dL crossed over, indicating a violation of the proportional hazard assumption. Therefore, we could not thoroughly assess the relative risk between these two categories.

## 5 CONCLUSION

Our study identified a significant increase in the risk of all‐cause mortality as FBG levels exceeded 125 mg/dL in PD patients with diabetes. Furthermore, the risk of cardiovascular mortality showed a substantial correlation with FBG levels compared with other causes of death. These findings provide valuable information for clinicians to manage blood glucose levels in PD patients with diabetes.

## AUTHOR CONTRIBUTIONS

Hyeon Seok Hwang, Ju Young Moon, Kyung Hwan Jeong, and Gang Jee Ko conceived and designed the study. Hyeon Seok Hwang, Gang Jee Ko, and Yun Jin Choi participated in formal analysis and data curation. So Jin Lim, and Hyeon Seok Hwang wrote the manuscript. So Jin Lim, Hyeon Seok Hwang, Ju Young Moon, Kyung Hwan Jeong, and Gang Jee Ko reviewed and edited the manuscript. All authors read and approved the final manuscript. The corresponding author attests that all listed authors meet the authorship criteria and that no others meeting the criteria have been omitted.

## CONFLICT OF INTEREST STATEMENT

The authors declare no conflicts of interest.

## Supporting information


**Figure S1.** Flow chart for patient enrollment. PD, peritoneal dialysis; FBG, fasting blood glucose.

## Data Availability

The datasets used and/or analyzed during the current study are available from the corresponding authors on reasonable request.
