# Supplementary material for: Fasting blood glucose level and risk of all‐cause and cause‐specific mortality in peritoneal dialysis patients
Source: J Diabetes. 2024 Sep 12;16(9):e13601. doi: 10.1111/1753-0407.13601 (PMC11391383; doi:10.1111/1753-0407.13601)
Supplement: Supplementary file 1 — Figure S1. Flow chart for patient enrollment. PD, peritoneal dialysis; FBG, fasting blood glucose. [file JDB-16-e13601-s001.docx]

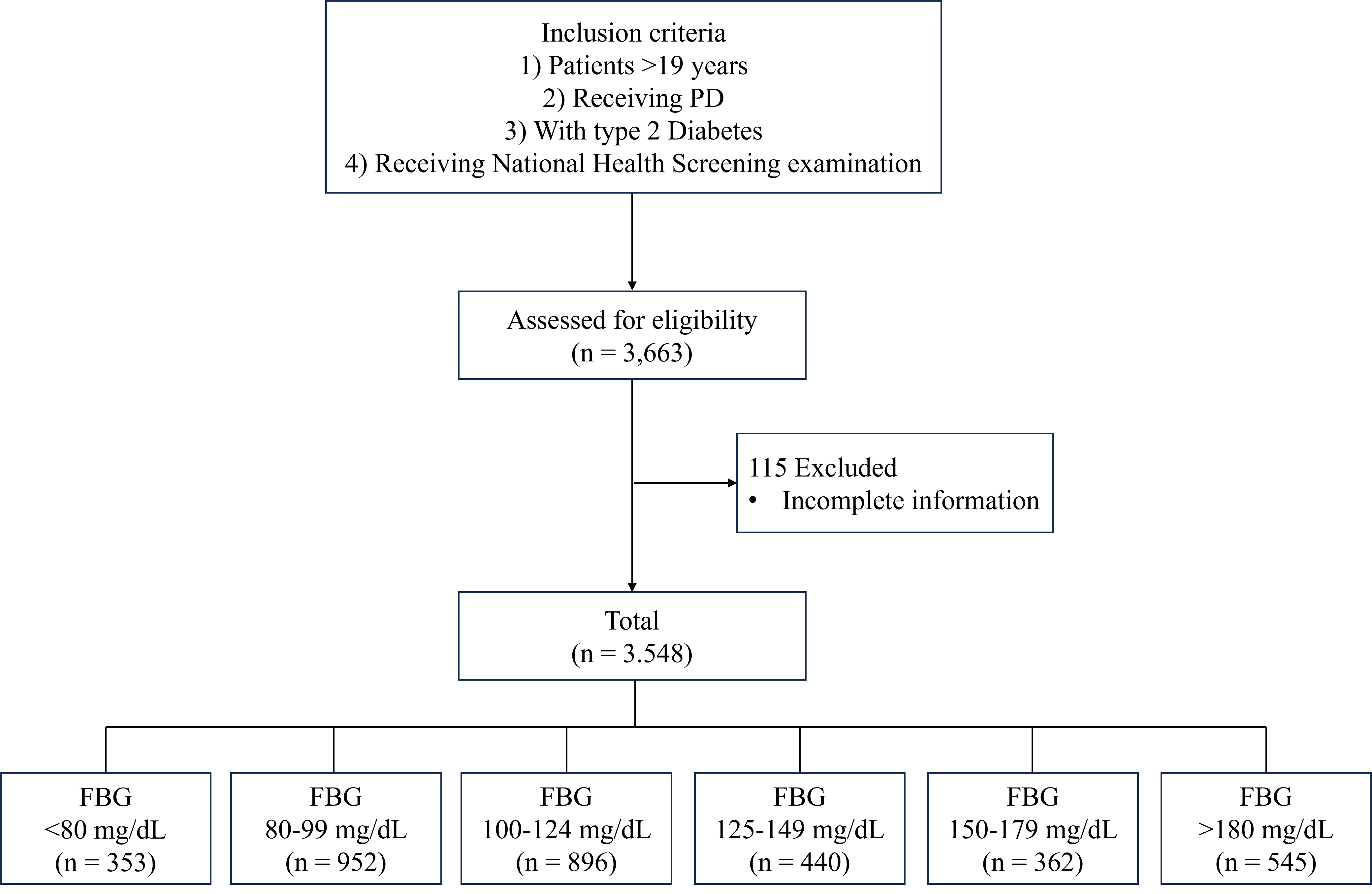


**Supplementary Figure 1.** Flow chart for patient enrollment. PD, peritoneal dialysis; FBG, fasting blood glucose.
